# Electrical Stimulation Prevents Preferential Skeletal Muscle Myosin Loss in Steroid-Denervation Rats

**DOI:** 10.3389/fphys.2018.01111

**Published:** 2018-08-10

**Authors:** Takashi Yamada, Koichi Himori, Daisuke Tatebayashi, Ryotaro Yamada, Yuki Ashida, Tomihiro Imai, Masayuki Akatsuka, Yoshiki Masuda, Keita Kanzaki, Daiki Watanabe, Masanobu Wada, Håkan Westerblad, Johanna T. Lanner

**Affiliations:** ^1^Graduate School of Health Sciences, Sapporo Medical University, Sapporo, Japan; ^2^Department of Intensive Care Medicine, Sapporo Medical University, Sapporo, Japan; ^3^Faculty of Health Science and Technology, Kawasaki University of Medical Welfare, Kurashiki, Japan; ^4^School of Life Sciences, La Trobe University, Melbourne, VIC, Australia; ^5^Graduate School of Integrated Arts and Sciences, Hiroshima University, Higashihiroshima, Japan; ^6^Department of Physiology and Pharmacology, Karolinska Institutet, Stockholm, Sweden

**Keywords:** muscle weakness, myosin loss, myofibrillar dysfunction, oxidative stress, electrical stimulation

## Abstract

Severe muscle weakness concomitant with preferential depletion of myosin has been observed in several pathological conditions. Here, we used the steroid-denervation (S-D) rat model, which shows dramatic decrease in myosin content and force production, to test whether electrical stimulation (ES) treatment can prevent these deleterious changes. S-D was induced by cutting the sciatic nerve and subsequent daily injection of dexamethasone for 7 days. For ES treatment, plantarflexor muscles were electrically stimulated to produce four sets of five isometric contractions each day. Plantarflexor *in situ* isometric torque, muscle weight, skinned muscle fiber force, and protein and mRNA expression were measured after the intervention period. ES treatment partly prevented the S-D-induced decreases in plantarflexor *in situ* isometric torque and muscle weight. ES treatment fully prevented S-D-induced decreases in skinned fiber force and ratio of myosin heavy chain (MyHC) to actin, as well as increases in the reactive oxygen/nitrogen species-generating enzymes NADPH oxidase (NOX) 2 and 4, phosphorylation of p38 MAPK, mRNA expression of the muscle-specific ubiquitin ligases muscle ring finger-1 (MuRF-1) and atrogin-1, and autolyzed active calpain-1. Thus, ES treatment is an effective way to prevent muscle impairments associated with loss of myosin.

## Introduction

Decreased muscle mass is an obvious cause of muscle weakness, but it is becoming increasingly clear that impairments intrinsic to the muscle fibers often make an important contribution to the loss of muscles strength (Reid and Moylan, 2011). For instance, a selective loss of the motor protein myosin has been found to have a key role in the reduction in maximal force production in a variety of pathophysiological conditions, including critical illness myopathy (Friedrich et al., 2015), cancer cachexia (Acharyya et al., 2004; Ochala and Larsson, 2008), chronic obstructive pulmonary disease (Ottenheijm et al., 2005), rheumatoid arthritis (Yamada et al., 2009), as well as in aging (D’Antona et al., 2003). The exact mechanisms underlying the myosin loss are not clear, but reduced mechanical stress and increased glucocorticoid signaling are considered to have important roles (Rouleau et al., 1987; Larsson et al., 2000; Borina et al., 2010; Brocca et al., 2015; Minetto et al., 2015).

Prolonged increases in production of reactive oxygen and nitrogen species (ROS/RNS) are implicated in several muscle pathologies. Studies on denervated skeletal muscle show activation of NADPH oxidase (NOX) (Bhattacharya et al., 2014) and nitric oxide synthase (NOS) (Suzuki et al., 2007), which increase superoxide and NO production, respectively. Additionally, increased ROS/RNS production was observed in skeletal muscle from rats with glucocorticoid-induced myopathy (Konno, 2005). Increased ROS/RNS has been shown to directly depress myofibrillar function in several pathophysiological conditions (Supinski and Callahan, 2007). Moreover, oxidative modifications of cellular proteins markedly accelerates their rate of degradation by, e.g., activation of calpains (Friedrich et al., 2018) and increases in the muscle-specific E3 ligases muscle ring finger-1 (MuRF-1) and atrogin-1, which are part of the ubiquitin-proteasome system (Grune et al., 2003; Betters et al., 2004; Smuder et al., 2010; Powers et al., 2016). Signaling via the p38 mitogen-activated protein kinase (MAPK) pathway has been proposed to mediate oxidative stress-induced atrogin-1 expression and ubiquitin-conjugating activity in skeletal muscle (Li et al., 2005; Jin and Li, 2007).

Electrical stimulation (ES) is used in the clinic as a rehabilitation/training method (Maffiuletti, 2010). Interestingly, ES training was shown to prevent the upregulation of atrogin-1 expression in denervated rat muscles (Lima et al., 2009). Moreover, we have recently reported that ES training prevents muscle dysfunction and oxidative stress in adjuvant-induced arthritis rats (Himori et al., 2017). However, the cellular and molecular mechanisms behind beneficial effects of ES on muscle function remain unclear.

In the present study we used the steroid-denervation (S-D) rat model, where animals were exposed to a combination of a pharmacological glucocorticoid treatment and surgical denervation (Rich et al., 1998; Kraner et al., 2011; Friedrich et al., 2018). Some S-D rats were exposed to daily ES treatment. We hypothesized that ES treatment would counteract the decline in muscle contractile function in S-D rats. In agreement with our hypothesis, ES treatment limited the weakness in S-D muscles, and we then went on to study tentative underlying mechanisms with focus on myosin loss and increases in ROS/RNS production.

## Materials and Methods

### Ethical Approval

All the experimental procedures were approved by the Committee on Animal Experiments of Sapporo Medical University (No. 16-076). Animal care was in accordance with institutional guidelines. A total of 24 rats (Sankyo Labo Service, Sapporo, Japan) were used in these experiments. For *in situ* muscle experiments, rats were anesthetized with 2% inhaled isoflurane to reach a stable anesthetic plane with consistent breathing rate and no response to toe pinch. At the end of the experiment, rats were killed by rapid cervical dislocation under isoflurane anesthesia and muscles were subsequently isolated.

### Induction of Steroid-Denervation

Male Wistar rats (9 weeks old, *n* = 24) were supplied by Sankyo Labo Service (Sapporo, Japan) and were randomly assigned into control (CNT) (*n* = 12) and S-D (*n* = 12) groups. Rats were given food and water ad libitum and housed in an environmentally controlled room (24 ± 2°C) with a 12-h light–dark cycle. S-D was induced by a combination of denervation and dexamethasone treatment (Kraner et al., 2012). Rat muscles were bilaterally denervated by removing a 10-mm segment of the sciatic nerve under 2% isoflurane anesthesia. Dexamethazone (5 mg/kg) was dissolved in saline at 2 mg/ml and injected intraperitoneally starting on the day of denervation and continuing for 7 days. Saline was injected in the control animals.

### Electrical Stimulation

**FIGURE 1:**
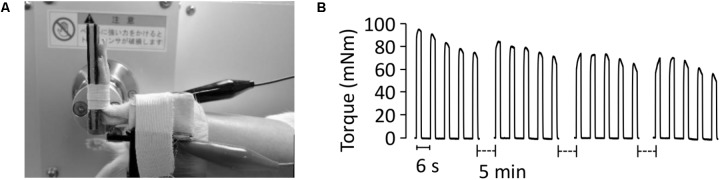
Procedure for electrical stimulation treatment. Under anesthesia, the animal was placed in a supine position and the left leg was attached to a footplate connected to a force transducer, with the right leg serving as control. The foot was placed at 0° angle of plantar flexion. The plantarflexor muscles were stimulated using a pair of surface electrodes on the skin **(A)**. The torque produced in response to the electrical stimulation was measured **(B)**. Each session consisted of 4 sets of 5 isometric contractions at 5-min intervals and was carried out every day for 7 days.

In both S-D and control animals, one side was subjected to the ES protocol and the other side served as non-ES control. Throughout the ES treatment sessions, rats were anesthetized by isoflurane inhalation. Rats were placed supine on a platform and their left foot was secured in a foot plate connected to a torque sensor (S-14154, Takei Scientific Instruments) at an angle of 0° plantarflexion (**Figure 1A**). Plantarflexor muscles, including the gastrocnemius, the plantaris, and the soleus muscles, were stimulated using a pair of surface electrodes (BlueSensor, Ambu, surface area of 0.785 cm^2^), which were strapped by tape to the posterior surface of the calf. ES training with long pulse duration has been shown to effectively improve force production in denervated muscles (Ashley et al., 2007). Thus, we set the stimulation parameters as follows: 10 ms monophasic rectangular pulse, 45V, 50 Hz. Each ES session consisted of 4 sets of 5 isometric contractions (2-s contraction given every 6 s) at 5-min intervals (**Figure 1B**). ES training was carried out for 7 consecutive days in both S-D and control groups and was initiated immediately after completion of the final dexamethasone injection in the S-D animals.

*In situ* plantar flexor torque was measured before the final ES training session. These measurements were performed with short (0.5 ms) current pulses to ensure that only one action potential was triggered by each pulse. The stimulation frequency ranged from 1 to 120 Hz and 600 ms contractions were produced at 1-min intervals. Control experiments were performed where the stimulation current was gradually increased until *in situ* torque no longer increased. To ensure that stimulation was supramaximal, a current 20% larger than that giving maximum *in situ* torque was used throughout experiments. About 24 h after the final ES training session, rats were killed by cervical dislocation under isoflurane anesthesia and the plantarflexor muscles were excised from each animal.

### Measurement of Ca^2+^-Activated Force in Skinned Fibers

Chemically skinned fibers were prepared according to Mollica et al. (2012) with some modifications. Gastrocnemius muscle was pinned out at resting length under paraffin oil that was kept at 4°C. Segments of single-skinned fibers were dissected under a stereo-microscope. A total of 4–6 skinned fibers were obtained from one whole muscle. The skinned fiber was connected to a force transducer (Muscle tester, World Precision Instruments) and then incubated with a *N*-2-hydroxyethylpiperazine-*N*^′^-2-ethanesulfonic acid (HEPES) buffered solution (see below) containing 1% (vol/vol) Triton X-100 for 10 min in order to remove membranous structures. Fiber length was adjusted to optimal length (2.5 μm) by laser diffraction as described previously (Allen and Kurihara, 1982) and the contractile properties were measured at room temperature (24°C).

All solutions were prepared as described in detail elsewhere (Watanabe and Wada, 2016). They contained (in mM) 36 Na^+^, 126 K^+^, 90 HEPES, 8 ATP and 10 creatine phosphate, and had a pH of 7.09–7.11 at 24°C. The free Mg^2+^ concentration was set at 1.0 mM. The maximum Ca^2+^ solution additionally contained 49.5 mM Ca-EGTA and 0.5 mM free EGTA, whereas the relaxation solution contained 50 mM free EGTA. Force-pCa (-log free Ca^2+^ concentration) curves were established with various pCa solutions (pCa 6.4, 6.2, 6.0, 5.8, 5.6, 5.4, and 4.7) prepared by mixing the maximum Ca^2+^ solution and the relaxation solution in appropriate ratios according to the affinity constants reported by Moisescu and Thieleczek (1978). The contractile apparatus was directly activated by exposing the skinned fiber to various pCa solutions and force was measured. The cross-sectional area of fibers was calculated from measurements of their diameters. All skinned fibers were used to determine the maximum Ca^2+^-activated force per cross-sectional area and the pCa_50_ was defined as the pCa at the half-maximal force.

### Determination of MyHC/Actin Ratio and Fiber Type in Skinned Fiber

At the completion of the functional measurements, the individual skinned fiber segments were diluted with 10 μl of non-reducing Laemmli buffer (mM): urea, 4000; Tris, 250; 4% SDS (vol/vol); 20% glycerol (vol/vol); 0.02% bromophenol blue (wt/vol). In pilot experiments, we confirmed that the skinned fiber samples contain the remaining nondiffusible components (i.e., myofibrillar proteins), but not the diffusible (i.e., cytosolic) proteins (data not shown). To separate the myofibrillar proteins, sodium dodecyl sulfate-polyacrylamide gel electrophoresis was performed using a 4–15% Criterion Stain-Free gel (Bio-Rad, Philadelphia, PA, United States). Images of the gels were acquired using Stain-Free imager (Bio-Rad). The ratio of myosin heavy chain (MyHC) to actin was evaluated densitometrically using ImageJ^1^ (National Institute of Health, Bethesda, MD, United States). Then, the fiber type was determined by immunoblot using anti-fast type MyHC antibody (see the Section “Immunoblots”).

### Immunoblots

Immunoblots were performed using: anti-titin (9D10, Developmental Studies Hybridoma Bank), anti-fast type MyHC (ab91506, Abcam), anti-actin (A4700, Sigma), anti-troponin (Tn) T (T6277, Sigma), anti-TnI (4002, Cell Signaling), anti-ryanodine receptor (RyR) 1 (MA3-925, Thermo), anti-dihydropyridine receptor (DHPR) (ab2864, Abcam), anti-sarcoplasmic endoplasmic reticulum Ca^2+^-ATPase (SERCA1) (MA3-911, Thermo), anti-SERCA2 (MA3-919, Thermo), anti-NOX2/gp91^phox^ (ab31092, Abcam), anti-NOX4 (ab133303, Abcam), anti-nNOS (610308, BD Biosciences), anti-endotherial NOS (eNOS) (610296, BD Biosciences), anti-glyceraldehyde 3-phosphate dehydrogenase (GAPDH) (010-25521, Wako), anti-total p38 MAPK (9202, Cell Signaling), and anti-p-p38 MAPK (Thr180/Tyr182) (4511, Cell Signaling).

To extract whole muscle proteins, muscle pieces were homogenized in ice-cold homogenizing buffer (40 μl/mg wet wt) consisting of (mM): Tris-maleate, 10; NaF, 35; NaVO_4_, 1; 1% Triton X 100 (vol/vol), and 1 tablet of protease inhibitor cocktail (Roche) per 50 ml. The protein content was determined using Bradford’s (1976) assay. Aliquots of the whole muscle homogenates (20 μg) were diluted with SDS-sample buffer (mM): Tris–HCl, 62.5; 2% SDS (wt/vol); 10% glycerol (vol/vol); 5% 2-mercaptoethanol (vol/vol); 0.02% bromophenol blue (wt/vol). Proteins were separated on 4–15% Criterion TGX Stain-Free gels (Bio-Rad). Gels were imaged (Bio-Rad Stain-Free imager) and the ratio of MyHC to the total muscle proteins was measured by using Image Lab Software (Bio-Rad). Then, proteins were transferred onto polyvinylidine fluoride membranes. Membranes were blocked in 3% (wt/vol) non-fat milk, Tris-buffered saline containing 0.05% (vol/vol) Tween-20, followed by incubation with primary antibody, made up in 1% (wt/vol) bovine serum albumin overnight at 4°C. Membranes were then washed and incubated for 1 h at room temperature (∼24°C) with secondary antibody (1:5000, donkey-anti-rabbit or donkey-anti-mouse, Bio-Rad). Images of membrane were collected following exposure to chemiluminescence substrate (Millipore) using a charge-coupled device camera attached to ChemiDOC MP (Bio-Rad), and Image Lab Software was used for detection as well as densitometry.

### Quantitative Real-Time PCR

Real-time PCR was used to quantify the mRNA levels for atrogin-1 and MuRF-1 in frozen gastrocnemius muscle tissue. Briefly, total RNA was isolated from muscle samples using RNeasy Fibrous Tissue Mini Kit (Qiagen) according to manufacturer’s instructions. Following isolation, the RNA was quantified using UV spectrophotometry (Thermo Scientific Nanodrop Light). Total RNA was reverse-transcribed to cDNA using Prime Script RT Reagent Kit (Takara). Synthesized cDNA was then amplified on the Applied Biosystems 7500 with Premix Ex Taq kit^TM^ (Takara). The following Taqman Probes (Applied Biosciences^TM^) were used: rat atrogin-1 (Ebxo32, Rn00591730_m1), rat muscle RING-Finger protein 1 (MuRF-1) (Trim63, Rn00590197_m1), and rat GAPDH (Rn01775763_g1). All samples were run in duplicate. Relative amounts of target mRNA was determined using the comparative threshold cycle method (ΔΔ*C*_T_). Expression of target genes was normalized to the corresponding expression level of GAPDH.

### Autolysis of Calpain-1

Muscle pieces of approximately 100 mg were diluted in nine volumes (mass/vol) of ice-cold homogenizing buffer (mM): EDTA, 5; EGTA, 5; Tris, 20; 0.001% (mass/vol) pepstatin A, 0.001% (mass/vol) 4-(2-aminoethyl)-benzenesulfonyfluoride (AEBF), 1 mM dithiothreitol (DTT), and 0.5 mM phenylmethylsulfonylfluoride (pH 7.4) and homogenized on ice using a hand-held glass homogenizer. Muscle proteins (20 μg) were separated on a 7% SDS-polyacrylamide gel and immunoblotting was performed using anti-calpain-1 antibody (C0355, Sigma) as described previously (Kanzaki et al., 2014). The amount of autolyzed calpain-1 was expressed as a percentage of total calpain in the same muscle sample.

### Statistics

Data are presented as mean ± SEM. Student’s unpaired *t*-test was used to detect differences in body weight between S-D and CNT rats. A two-way ANOVA was performed to evaluate the influence of S-D and ES. A Tukey–Kramer *post hoc* test was used when significant differences were determined using ANOVA. A *P*-value < 0.05 was regarded as statistically significant.

## Results

### Body and Muscle Weights

The body weights of the S-D rats were significantly lower than those of the control group (**Table 1**). In addition, absolute weight for plantarflexor muscles, including the soleus, the plantaris, and the gastrocnemius muscles was ∼50% lower in the S-D rats than in controls (**Table 1**). ES treatment had no effect in control rats, whereas in S-D rats it attenuated the decrease in muscle weight for the fast-twitch plantaris and gastrocnemius muscles, but not for the slow-twitch soleus muscles.

**Table 1:** Body and muscle weight of control and steroid-denervation rats.

	*n*	Body (g)	SOL (mg)	PLA (mg)	GAS (mg)	Whole (mg)
**CNT**	6	234 ± 7	89 ± 3	221 ± 5	1150 ± 36	1461 ± 43
**CNT+ES**	6		86 ± 2	224 ± 7	1137 ± 43	1447 ± 43
**S-D**	6	182 ± 4^∗^	54 ± 3^∗^	113 ± 2^∗^	602 ± 18^∗^	769 ± 21^∗^
**S-D+ES**	6		51 ± 2^∗^	145 ± 4^∗,#^	742 ± 21^∗,#^	938 ± 23^∗,#^

*Values are means ± SEM. CNT, control; S-D, steroid-denervation; ES, electrical stimulation; *n*, number of samples; SOL, soleus; PLA, plantaris; GAS, gastrocnemius; Whole, summation of SOL, PLA, and GAS. ^∗^*P* < 0.05 vs. CNT, ^#^*P* < 0.05 vs. SD*.

### ES Treatment Improves Contractile Function in S-D Muscles

**FIGURE 2:**
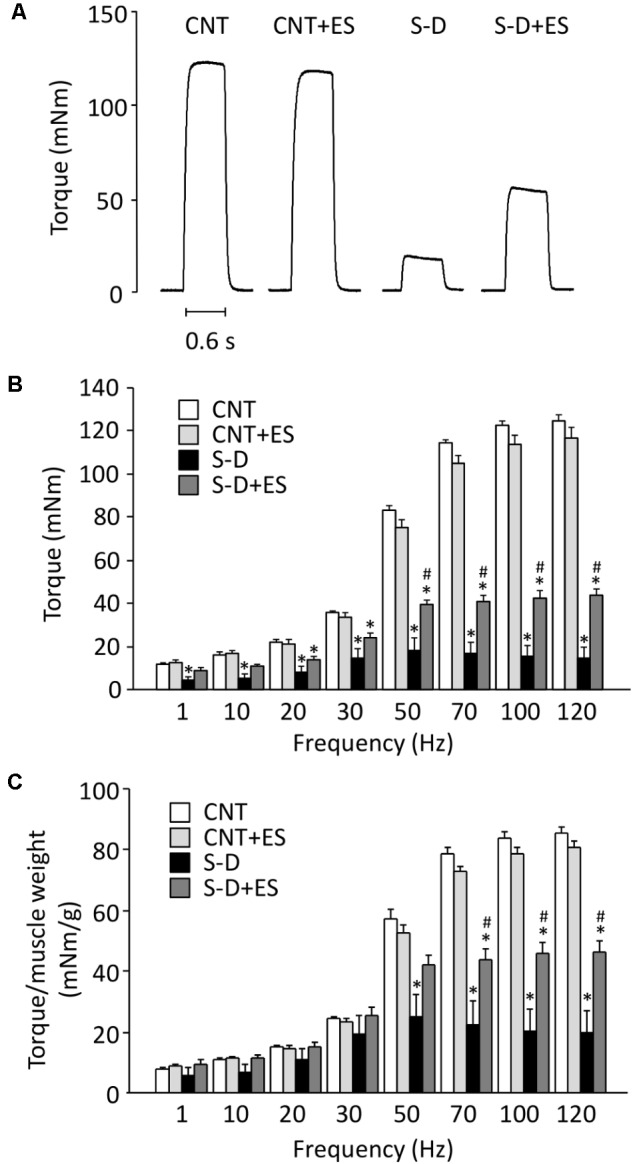
ES treatment ameliorates the decrease in *in situ* plantarflexor torque in S-D rats. Representative original records of 120 Hz tetanic torque in plantarflexor muscles from control (CNT) and steroid-denervation (S-D) rats with or without ES treatment **(A)**. Torque–frequency relationship **(B)**. Torque–frequency relationship normalized to the whole muscle weight for plantarflexors **(C)**. Bars show the mean and SEM results from 5 to 6 muscles per group. ^∗^*P* < 0.05 vs. CNT, ^#^*P* < 0.05 vs. S-D.

The S-D induced a severe depression in *in situ* plantarflexor torque (**Figures 2A,B**). Although less marked, this torque depression was still present after normalizing to the whole muscle weight (**Figure 2C**), which implies that it was due to the combined effect of decreased muscle cross-sectional area and defective activation and/or contractile function of the muscle fibers. ES treatment significantly increased the *in situ* torque in S-D rats, whereas it had no effect in control rats.

**FIGURE 3:**
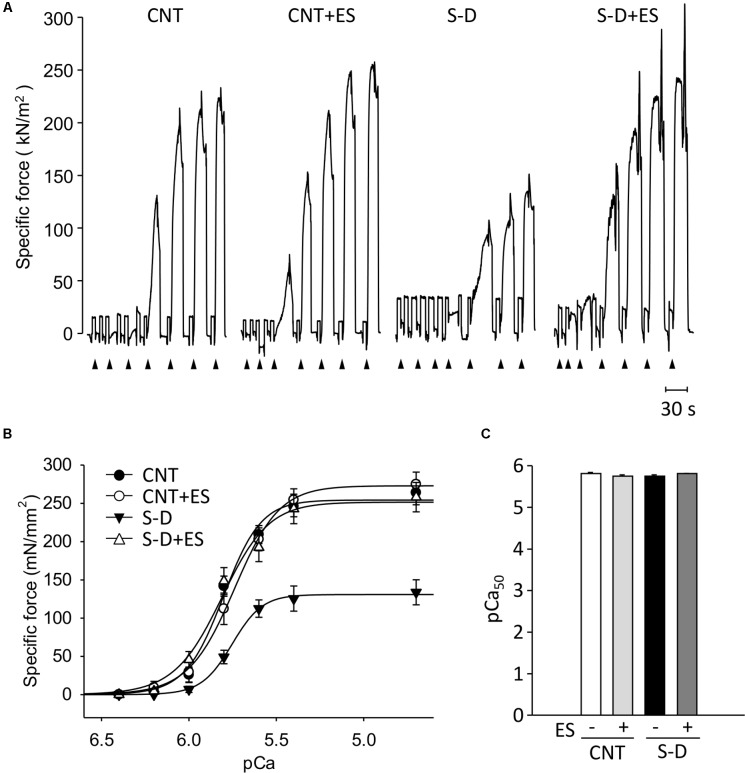
ES treatment prevents the force depression in skinned muscle fibers from S-D rats. Representative original records of Ca^2+^-activated force in chemically skinned fibers from gastrocnemius muscles of control (CNT) and steroid-denervation (S-D) rats with or without ES treatment. Fibers were exposed to solutions at progressively higher free Ca^2+^ concentration: pCa 6.4, 6.2, 6.0, 5.8, 5.6, 5.4, and 4.7 **(A)**. Specific force–frequency relationship **(B)** and pCa_50_ **(C)**. Data presented as mean and SEM from 14 to 18 fibers per group.

**Figure 3A** shows the typical traces of Ca^2+^-activated force in skinned fibers from each groups. Following seven days of ES, the diameters of the fibers in S-D (35.5 ± 1.7 μm, *n* = 14) and S-D+ES (35.3 ± 1.1 μm, *n* = 18) groups were significantly lower than those of the CNT (45.7 ± 1.6 μm, *n* = 17) and CNT+ES (46.0 ± 1.6 μm, *n* = 15) groups. Force-pCa curves constructed from mean data show that the maximum Ca^2+^-activated force (P_max_) was reduced by ∼50% in fibers from S-D muscles relative to those of control muscles (131 ± 73 vs. 254 ± 29 mN/mm^2^, *P* < 0.05) (**Figure 3B**). Intriguingly, ES treatment completely prevented the S-D-induced decrease in skinned fiber specific force. There was no difference in pCa_50_ between the groups (**Figure 3C**).

### ES Treatment Prevents the Myosin Loss in S-D Muscles

**FIGURE 4:**
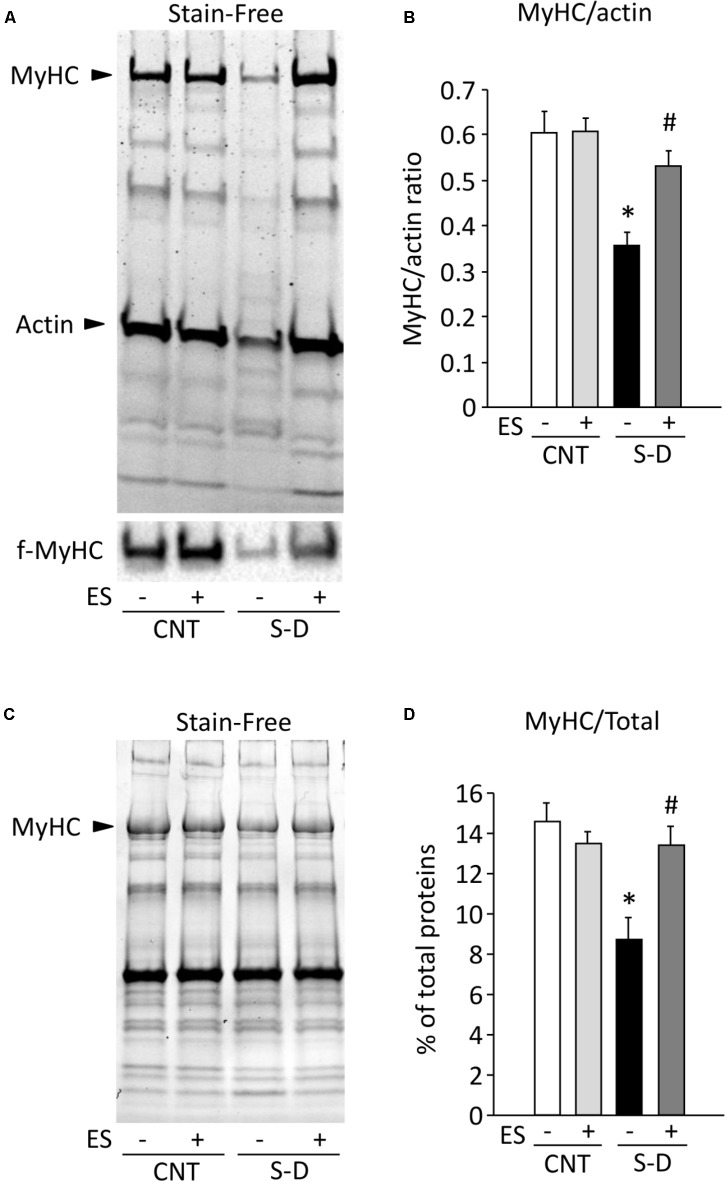
ES treatment prevents the myosin loss in S-D muscles. Representative stain-free gel of myofibrillar proteins and immunoblots for fast type (f)-MyHC in skinned fibers in control (CNT) and steroid-denervation (S-D) rats with or without ES treatment **(A)**. The ratio of MyHC to actin **(B)**. Bars show the mean and SEM results from 14 to 18 fibers per group. ^∗^*P* < 0.05 vs. CNT, ^#^*P* < 0.05 vs. S-D. Representative stain-free gel of the total proteins in whole muscle homogenates **(C)**. The expression levels of MyHC normalized to the total proteins **(D)**. Bars show the mean and SEM results from 5 to 6 muscles per group. ^∗^*P* < 0.05 vs. CNT, ^#^*P* < 0.05 vs. S-D.

The rat gastrocnemius muscle contains predominantly fast-twitch muscle fibers (Yoshihara et al., 2016) and immunoblot analysis performed with the anti-fast type MyHC antibody revealed that all skinned fibers analyzed in this study were fast-twitch fibers (**Figure 4A**). The decrease in P_max_ in skinned S-D muscle fibers can, in principle, be due to a decreased number of force producing myosin cross-bridges and/or impaired cross-bridge function with decreased force per cross-bridge. To distinguish between these two alternatives, we measured the ratio of MyHC to actin in skinned fibers and observed ∼40% decrease in S-D muscles, which was fully prevented by ES treatment (**Figures 4A,B**). Similarly, in the whole muscle homogenates, MyHC content was reduced by about 40% in S-D muscles, which was reversed by ES treatment (**Figures 4C,D**). Thus, these changes in MyHC expression combined with results from the skinned fiber experiments suggest that a decreased number of myosin cross-bridges contribute to the decrease in P_max_.

**FIGURE 5:**
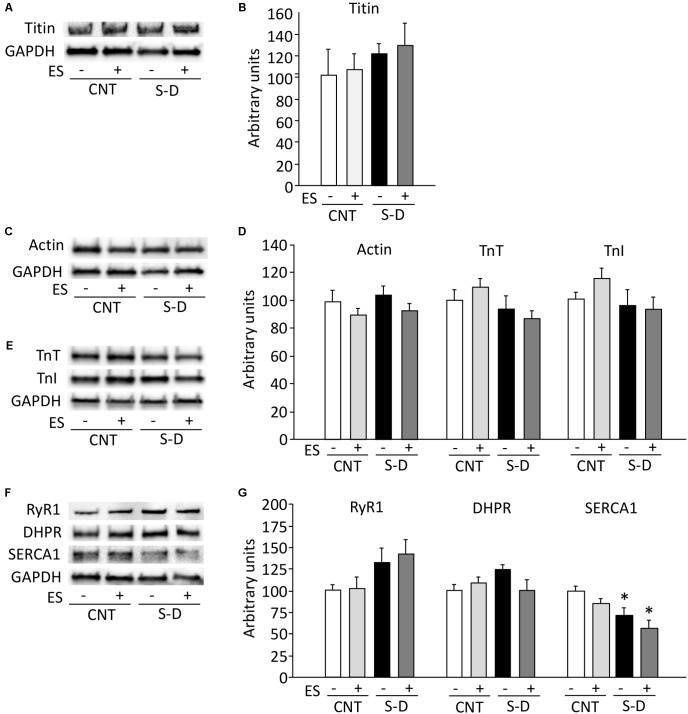
Expressions of thin filament and sarcoplasmic reticulum Ca^2+^ handling proteins are unaltered in S-D muscles. Representative immunoblots of myosin filament-linked protein titin **(A)**, thin filament proteins actin **(C)**, troponin (Tn) T, TnI **(D)**, sarcoplasmic reticulum Ca^2+^ handling proteins ryanodine receptor (RyR) 1, DHPR-α2 subunit, and sarcoplasmic reticulum Ca^2+^-ATPase (SERCA) 1 **(F)**. The expression levels of titin **(B)**, actin, TnT, TnI **(E)**, RyR1, DHPR, and SERCA1 **(G)** normalized to the glyceraldehyde-3-phosphate dehydrogenase (GAPDH) content. Results are expressed as a percentage of CNT value. Bars show the mean and SEM results from 5 to 6 muscles per group. ^∗^*P* < 0.05 vs. CNT.

To investigate whether the decrease in MyHC expression was part of a general decline in myofibrillar protein expression or specific to MyHC, we measured the expression of other myofibrillar proteins. The expression of the myosin filament-linked protein titin was not affected by S-D or ES treatment (**Figures 5A,B**). Neither the expression of the major thin filament protein actin nor the expression of the regulatory proteins troponin T (TnT) and troponin I (TnI), which have been linked to decreased specific force in previous studies (Ingalls et al., 1998; Adams et al., 2008), was affected by S-D or ES treatment (**Figures 5C–E**).

The decrease in *in situ* torque in S-D muscles was larger than the decrease in skinned fiber force and, in contrast to skinned fiber force and MyHC expression, it was not fully reversed by ES treatment. This implies that the force decrease in S-D muscles also involved impaired activation of the contractile machinery. Therefore, we measured the protein expression of three proteins central to the sarcoplasmic reticulum (SR) Ca^2+^ release: the SR Ca^2+^ release channel, the ryanodine receptor 1 (RyR1), the t-tubular voltage sensor, the DHPR; the fast-twitch fiber isoform of the SR Ca^2+^ pump (SERCA1; the slow-twitch isoform, SERCA2, was not detected in either group). RyR1 and DHPR protein expressions were not affected by either S-D or ES treatment, whereas the expression of SERCA1 was significantly decreased in S-D muscles and this decrease was not prevented by ES treatment (**Figures 5F,G**).

### Protein Expressions of ROS/RNS Producing Proteins Are Upregulated in S-D Muscles

**FIGURE 6:**
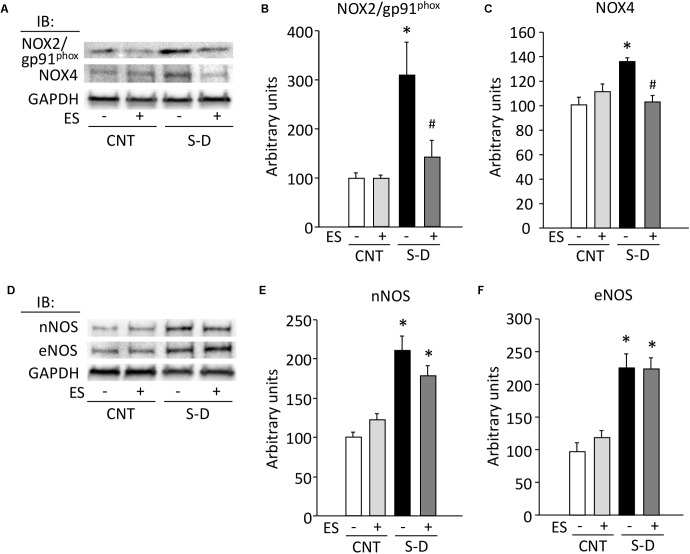
Expressions of redox-related proteins are upregulated in S-D muscles. Representative immunoblots illustrating the levels of NOX (NOX2/gp91^phox^), NOX4 **(A)**, neuronal NOS (nNOS), and endotherial NOS (eNOS) **(D)** of gastrocnemius muscles in control (CNT) and steroid-denervation (S-D) rats with or without ES treatment. The levels of NOX2/gp91^phox^ **(B)**, NOX4 **(C)**, nNOS **(E)**, and eNOS **(F)** expressions were normalized to the glyceraldehyde-3-phosphate dehydrogenase (GAPDH) content. Bars show the mean and SEM results from 5 to 6 muscles per group. ^∗^*P* < 0.05 vs. CNT, ^#^*P* < 0.05 vs. S-D.

The expressions of the NOX isoforms NOX2/gp91^phox^, NOX4, as well as the neuronal and endothelial NOS (nNOS, eNOS) were significantly increased in S-D muscles (**Figure 6**), whereas inducible NOS (iNOS) was not detected in any of the conditions. ES treatment prevented the upregulation of NOX2/gp91^phox^ and NOX4, whereas it had no effect on the S-D-induced increases in nNOS and eNOS.

### ES Treatment Limits the Increase in Phosphorylation of p38 MAPK and mRNA Expression of Ubiquitin Ligases in S-D Muscles

**FIGURE 7:**
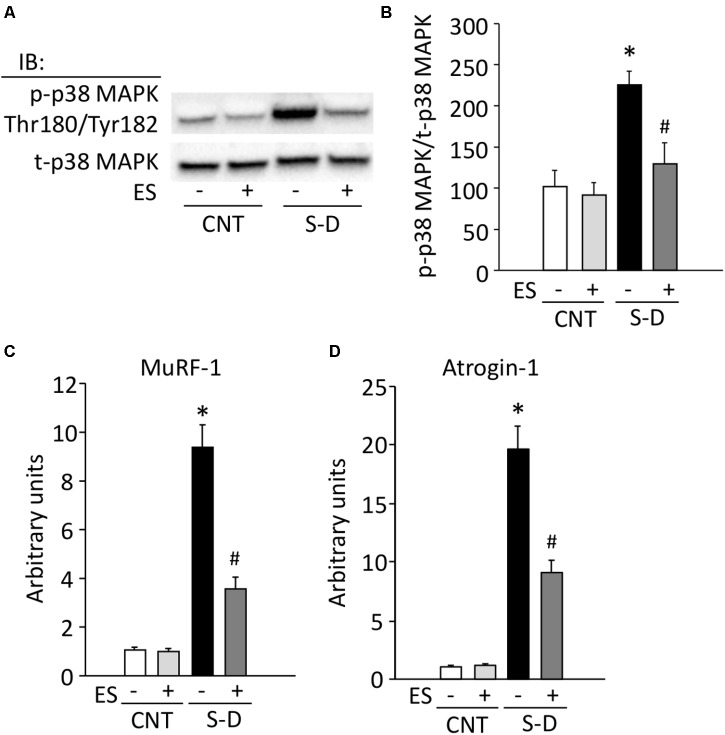
ES treatment limits the increase in phosphorylation of p38 MAPK and mRNA expression of ubiquitin ligases in S-D muscles. **(A)** Representative Western blots illustrating the total and p-p38 MAPK (Thr180/Tyr182) of gastrocnemius muscles in control (CNT) and steroid-denervation (S-D) rats with or without ES treatment. **(B)** The expression levels of p-p38 MAPK was normalized to the total p38 MAPK. The expression levels of MuRF-1 **(C)** and atrogin-1 **(D)** mRNA were normalized to the glyceraldehyde-3-phosphate dehydrogenase (GAPDH) mRNA and expressed as fold change of the mean CNT value, which was set to 1. Bars show the mean and SEM results from 5 to 6 muscles per group. ^∗^*P* < 0.05 vs. CNT, ^#^*P* < 0.05 vs. S-D.

Increases in ROS/RNS have been shown to promote protein breakdown via activation of p38 MAPK signaling and the ubiquitin-proteasome system (Grune et al., 2003; Betters et al., 2004; Li et al., 2005; Jin and Li, 2007; Powers et al., 2016). Phosphorylation of p38 MAPK was about twofold higher in S-D muscles than in CNT muscles (**Figures 7A,B**). Moreover, compared to the CNT group, S-D induced a 9- and 19-fold increase in the mRNA expression of the muscle-specific ubiquitin ligases MuRF-1 and atrogin-1, respectively (**Figures 7C,D**). ES treatment significantly suppressed the phosphorylation of p38 MAPK and the expression levels of MuRF-1 and atrogin-1 mRNA in S-D muscles.

### ES Treatment Prevents the Activation of Calpain-1 in S-D Muscles

**FIGURE 8:**
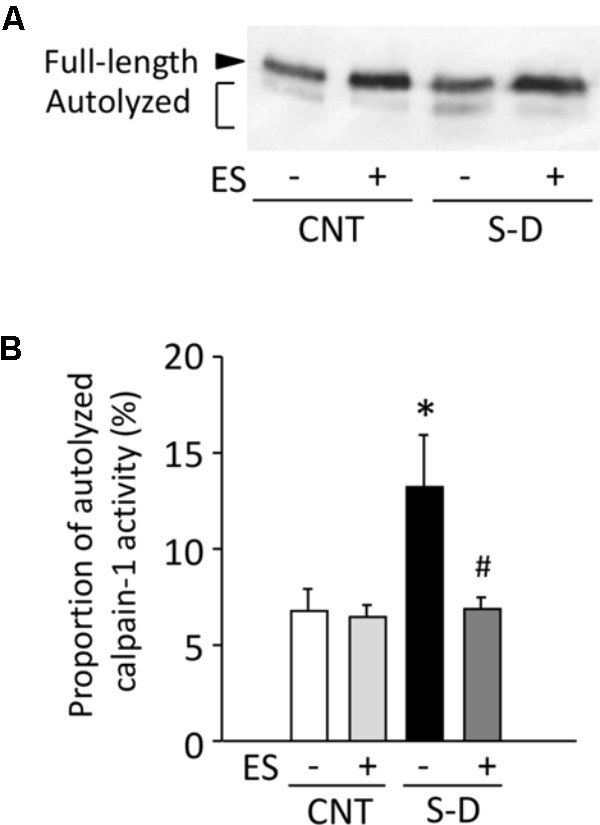
ES treatment prevents the activation of calpain-1 in S-D muscles. **(A)** Representative Western blots illustrating the autolysis of calpain-1 of gastrocnemius muscles in control (CNT) and steroid-denervation (S-D) rats with or without ES treatment. **(B)** The content of autolyzed calpain-1 was expressed as a percentage of the total calpain-1 in the same muscle sample. Bars show the mean and SEM results from 5 to 6 muscles per group. ^∗^*P* < 0.05 vs. CNT, ^#^*P* < 0.05 vs. S-D.

Calpain-mediated protein degradation has been linked to increased ROS/RNS production and oxidative protein modifications (Smuder et al., 2010). Ca^2+^ triggers an autolytic process in calpain-1 and reduces the [Ca^2+^] required for its activation from 400–800 to 50–150 μM (Goll et al., 2003). Full-length calpain-1 exists as an 80-kDa protein and can be autolyzed to proteins of 78 and 76 kDa. Immunoblot analysis showed that the amounts of autolyzed calpain-1 were elevated in S-D muscles and this was prevented by ES treatment (**Figures 8A,B**).

## Discussion

In skeletal muscle of S-D rats, we here show reduced *in situ* force production accompanied by decreases in maximum Ca^2+^-activated force and MyHC protein content. Importantly, the S-D-induced decreases in maximum Ca^2+^-activated force and MyHC protein content were fully prevented by daily treatment with ES, and the *in situ* force production was improved although not to the level of control muscle.

**FIGURE 9:**
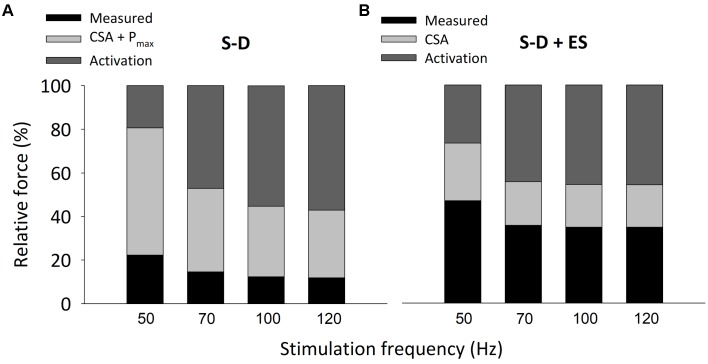
The estimated activation failure contributing to the decreased *in situ* plantarflexor torque was not prevented by E-S treatment. Estimates of relative force components underlying the difference between control and S-D **(A)** or S-D + ES **(B)** isometric torque at 50–120 Hz stimulation. The mean torque in control muscles (from **Figure 2A**) was set to 100% in each bar. Black part in bars represents mean torque in S-D and S-D + ES muscles, respectively (from **Figure 2A**). Light gray part in bars represent the fraction of force deficit that can be explained by the decrease in cross-sectional area (CSA; calculated from **Table 1**) and the decrease in maximum force-generating capacity (P_max_; from **Figure 3B**); note that P_max_ was not affected in S-D + ES muscle. Dark gray part in bars reflects the fraction of force deficit that cannot be explained by decreased myofibrillar force-generating capacity, which was assigned as activation failure.

Two components obviously contribute to the present S-D-induced reduction in isometric *in situ* plantarflexor force: muscle atrophy and decreased P_max_. The mean weight of the whole plantarflexor muscles in S-D rats was 52% of the control value (see **Table 1**). Assuming that S-D does not affect the muscle architecture, this corresponds to an equivalent decrease in muscle cross-sectional area and hence decreases in plantarflexor force production. Moreover, skinned fiber experiments revealed a reduction in maximum force per cross-sectional area (P_max_) in S-D muscle down to 53% of the control value (see **Figure 3**), which further reduces plantarflexor force production. The decrease in P_max_ can largely be explained by a decreased number of force-producing cross-bridges, since there was a specific decrease in MyHC protein expression down to 59% of the control (see **Figure 4**), i.e., a reduction of a magnitude similar to that of P_max_. The relative S-D-induced decrease in plantarflexor force was less prominent at low than at high stimulation frequencies (see **Figure 2B**). Thus, the decrease in plantarflexor force at 1–30 Hz stimulation can be fully explained by the combined effect of decreased muscle cross-sectional area and reduced P_max_. However, this is not the case at 50 Hz and higher stimulation frequencies. In **Figure 9A**, we use mean data of the *in vivo* plantarflexor torque (from **Figure 2B**), cross-sectional area (i.e., plantarflexor muscle weight from **Table 1**) and P_max_ (from **Figure 3**) to estimate the relative contribution of different components to the decreased force in S-D muscle. This assessment reveals that at 50 Hz, ∼20% of the force decrease in S-D muscles is not explained by the combined effect of decreased cross-sectional area and reduced P_max_, and this component increases to almost 60% at 120 Hz. This “unexplained” component would correspond to factors upstream of the contractile machinery, i.e., activation impairments.

**Figure 9B** shows estimates of components underlying the lower forces at 50–120 Hz stimulation in ES-treated S-D muscles vs. control muscles. ES-treated S-D whole plantarflexor muscles showed less decrease in weight than untreated S-D muscles, although their mean weight was still decreased to 64% of control muscles. Moreover, P_max_ and the MyHC content were not decreased in ES-treated S-D muscle. Although the *in vivo* plantarflexor torque was significantly larger in ES-treated than untreated S-D muscle, the smaller reduction in cross-sectional area and the absence of a decrease in P_max_ with ES treatment results in an activation failure component of similar size to that in untreated S-D muscle. Thus, these estimates indicate that ES treatment of S-D muscle does not protect against activation impairments.

The S-D-induced activation deficiency, as revealed by the estimates depicted in **Figure 9**, was observed at 50 Hz and higher stimulation frequencies. Thus, its characteristics are opposite to those of the prolonged low-frequency force depression frequently observed after strenuous exercise, where force is more depressed at low than at high-frequency stimulation (Allen et al., 2008b). A possible mechanism underlying the larger S-D induced activation deficiency at high stimulation frequencies is reduced SR Ca^2+^ storage, which would decrease the amount of Ca^2+^ available for release and thereby limit the increase in cytosolic [Ca^2+^] and force that can be achieved at high stimulation frequencies. In favor of this possibility, the protein expression of the SR Ca^2+^ pumps, SERCA1, was decreased both in untreated and ES-treated S-D muscles (see **Figure 5G**), and decreased SR Ca^2+^ uptake would limit SR Ca^2+^ storage. However, a recent study using rats exposed to prolonged intensive care unit-mimicking sedation and muscle paralysis shows severe muscle weakness with impaired SR Ca^2+^ release and decreased SERCA1 protein expression, but the amount of Ca^2+^ stored in the SR was not decreased as judged from an unaltered increase in cytosolic free [Ca^2+^] during tetani produced in the presence of caffeine, which facilitates SR Ca^2+^ release (Llano-Diez et al., 2016).

Another possible mechanism underlying the larger S-D induced activation deficiency at high stimulation frequencies is membrane hypo-excitability leading to deficient action potential generation and propagation, which would become more marked with increasing stimulation frequency (Allen et al., 2008a; Z’Graggen et al., 2011). This membrane dysfunction is most likely caused by a depolarization at rest combined with a hyperpolarizing shift in the voltage dependence of Na^+^ channel inactivation (Filatov and Rich, 2004). It might be that the ES training used in the present study (4 sets of 5 contractions) was too limited to effectively prevent the activation deficiency in S-D muscles. In fact, more than 200 contractions daily were required to maintain force production in chronically denervated rat extensor digitorum longus muscles (Dow et al., 2004). Moreover, the stimulation current might be too low in the S-D state, although it was supramaximal in the control state. Additionally, our stimulation frequency (50 Hz) reduces muscle wasting in fast-twitch, but not slow-twitch, muscles in S-D rats. For slow-twitch soleus muscle, a stimulation frequency of 20 Hz has been proven efficient to maintain muscle mass in denervated soleus muscles, which is a stimulation pattern that resembles the physiological activity of motoneurones (Gundersen et al., 1988). Accordingly, future studies are needed to determine the optimal ES protocol to prevent the *in situ* force depression in S-D rats.

In S-D muscles, we here show concomitant increases in the protein expression of superoxide-producing NOX2 and NOX4 as well as NO-producing nNOS and eNOS. While the increases in NOXs were prevented by ES treatment, the increases in NOSs were not. The p38 MAPK pathway has been implicated in mediating oxidative stress-induced protein breakdown via the ubiquitin-proteasome system (Li et al., 2005; Jin and Li, 2007). Our results show marked increases in the phosphorylation of p38 MAPK and the mRNA expression of the muscle-specific ubiquitin ligases MuRF-1 and atrogin-1 in S-D muscles and ES treatment significantly suppressed these increases. Calpain-mediated protein degradation has been linked to increased ROS/RNS production (Smuder et al., 2010). We observed an increased level of autolyzed active calpain-1 in S-D muscles and this increase was prevented by ES treatment. Thus, our results indicate that the specific degradation of MyHC in S-D muscles was mediated via activation of the ubiquitin-proteasome system and calpain-1 due to increased ROS produced by NOXs. Conversely, an isolated increase in RNS production by NOSs did not have a key role since ES treatment did not prevent the increases in NOSs expression. Moreover, our results suggest that while activation of the ubiquitin-proteasome system and calpain-1 has an essential role in the decrease in MyHC in S-D muscles, additional degrading systems are involved since ES treatment only partly counteracted the muscle atrophy.

## Conclusion

We here show that ES treatment is an effective way to reduce muscle impairments in the S-D rat. In S-D muscle, ES treatment fully prevented the decrease in myofibrillar force production due to decreased MyHC expression and partly prevented muscle atrophy, whereas it did not prevent the activation failure.

## Author Contributions

TY contributed to the conception and design of the study. TY, KH, TI, MA, YM, KK, DW, MW, HW, and JL participated in the analysis and interpretation of the data. TY, KH, DT, RY, YA, and KK were responsible for the data collection. TY, HW, and JL were involved in writing the manuscript and all the authors approved the final version. All the authors agreed to be accountable for all aspects of the work in ensuring that questions related to the accuracy or integrity of any part of the work were appropriately investigated and resolved. All the persons who were designated as authors and qualified for authorship were listed. All the experiments were performed at the Muscle Physiology Laboratory in the Graduate School of Health Sciences, Sapporo Medical University, Sapporo, Japan.

## Conflict of Interest Statement

The authors declare that the research was conducted in the absence of any commercial or financial relationships that could be construed as a potential conflict of interest.

## Footnotes

1 https://imagej.nih.gov/ij/
